# Posturography and dynamic pedobarography in lame dogs with elbow dysplasia and cranial cruciate ligament rupture

**DOI:** 10.1186/s12917-018-1435-y

**Published:** 2018-03-24

**Authors:** José M. Carrillo, Maria E. Manera, Mónica Rubio, Joaquin Sopena, Angelo Santana, José M. Vilar

**Affiliations:** 1 Departamento Medicina y Cirugía Animal, Cátedra García Cugat, Universidad CEU Cardenal Herrera, Valencia, Spain; 2 Departamento de Patología Animal, Instituto Universitario de Investigaciones Biomédicas y Sanitarias, Universidad de las Palmas de Gran Canaria, Arucas, Las Palmas, Spain; 3 Departamento de Matemáticas, Universidad de las Palmas de Gran Canaria, Las Palmas, Spain

**Keywords:** Pressure platform, Posturography, Pedobarography, Lameness, Dog, COP

## Abstract

**Background:**

The usefulness of studying posture and its modifications due to locomotor deficiencies of multiple origins has been widely proven in humans. To assess its suitability in the canine species, static posturography and dynamic pedobarography were performed on lame dogs affected with unilateral elbow dysplasia and cranial cruciate ligament rupture by using a pressure platform. With this objective, statokinesiograms and stabilograms, the percentage of pressure distribution between limbs, paw area, mean pressure, and peak pressure, were obtained from lame and sound dogs. These data were compared with Peak Vertical Force values originated from a force platform in the same recording sessions.

**Results:**

Significant differences were found in the parameters mentioned above between sound and lame dogs and limbs.

**Conclusions:**

Posturography and pedobarography are useful and reliable for the monitoring of fore and hindlimb lameness in dogs, providing a new set of parameters for lameness detection.

**Electronic supplementary material:**

The online version of this article (10.1186/s12917-018-1435-y) contains supplementary material, which is available to authorized users.

## Background

Osteoarthritis (OA) could affect up to 20% of the canine population [1]. OA represents 47% of musculoskeletal diseases, affecting 42.5% of hips, 18.5% of stifles, and 12.8% of elbow joints [2]. Although it could have a multifactorial etiology, OA appears mainly secondary to articular instabilities, which occur in elbow dysplasia (ED) and cranial cruciate ligament rupture (CCLR), respectively. ED is an inherited, complex syndrome that comprises medial compartment disease, osteochondritis dissecans, ununited anconeal process, and articular surface incongruity [3, 4]. The classic diagnosis is based on radiological signs and/or arthroscopy [5]. On the other hand, CCLR is one of the most frequent stifle lesions, often causing lameness in dogs [6, 7], and OA develops over time from these lesions [8]. Moreover, animals with CCLR are positive for tibial compression and cranial drawer tests. Radiographies under tibial compression with the animal in recumbency confirm the presence of CCLR by means of visualization of cranial displacement of the proximal end of the tibia with respect to the femoral condyles among other signs [9]. Lameness intensity in a walking animal could vary from obvious lameness to not supporting. Some authors describe it as a “toe-touching” gait [10].

The presence of poor agreement between clinical, radiological, or even arthroscopic signs [11] in lame dogs is producing a rapid development of kinematic and kinetic-based gait analysis techniques [12, 13], which could be a complementary and objective method of defining lameness in dogs [14, 15]. Posturography assesses the integrity of the balance system, and it is widely used in human medicine for the detection of musculoskeletal disorders [16]. The pathologic changes in posture are detected by means of recording the body’s center of pressure (COP) sway via statokinesiograms and/or stabilograms; statokinesiograms graphically represent the area in mm^2^ of an ellipse that includes 90% of the points registered during the COP sway in a 2-D space. On the other hand, stabilograms show specific COP migration in the X and Y axes. In this way, the better the stability, the smaller the value [17]. These changes determine an abnormal distribution of pressure within the paws during the support phase, which can be evaluated through pedobarography studies not only while standing still (static pedobarography) but also while walking (dynamic pedobarography) [18]. In addition, due to the elastic nature of the dog’s pads, paw area increases as pressure increases [19, 20].

Studies of pressure platforms are increasing in veterinary medicine, although the majority describe force-related data in healthy [20, 21] and lame dogs with CCLR [6], hip osteoarthritis [22], or dogs with total hip replacement [23]. More recently, pressure-dependent characteristics have started to be assessed as a valuable factor to assess lameness via symmetry index or static pedobarography [19, 24]. Regarding postural changes in lame dogs, only statokinesiograms have been included in a single report [19], and no studies involving dynamic pedobarography could be found.

In terms of limb function, Peak Vertical Force (PVF) is currently considered the gold standard test [25]; for that reason, the present study aims to obtain a series of postural and dynamic pedobarographic parameters that could objectively help detect lameness in OA dogs suffering from ED and CCLR. Validation of data was performed, comparing these results with PVF values simultaneously originated from a force platform. We hypothesize that lame, OA dogs have postural and pedobarographic changes when compared with sound dogs, as occurs when PVF has routinely been used as parameter for lameness assessment.

## Methods

### Animals

This study utilized 34 client-owned, adult dogs with similar conformation. The body weights of the enrolled dogs ranged from 30 to 44.6 kg, and the ages were 3 to 9 years. The control (sound, *n* = 10) group was formed by healthy dogs without previous clinical history of lameness. Two study groups were formed with dogs with unilateral ED (*n* = 12) and CCLR (n = 12). Gender and sexual status of all dogs, as well as each body condition score under WASAVA criteria [26] are showed in Table 1.

**Table 1:** Breed and gender distribution of 34 dogs included in this study. Body condition score and bioarth scale score are also provided for each dog

	Breed	Gender-status	BCS	BSS
Control				
1	Mixed	MN	7	2
2	Mixed	M	5	3
3	Labrador	FS	6	2
4	Pit bull	M	5	4
5	Rottweiler	F	6	2
6	Bull terrier	MN	6	2
7	Rottweiler	F	4	3
8	Mixed	FS	5	4
9	Husky	M	6	3
10	Pit bull	M	5	3
ED				
1	Chow-chow	F	4	26
2	Schnauzer	F	6	23
3	Mixed	MN	7	27
4	Labrador	M	3	18
5	Mixed	M	5	19
6	Weimaraner	FS	7	22
7	Mixed	MN	5	23
8	Alaskan m	FS	5	26
9	Labrador	F	4	30
10	Weimaraner	M	5	24
11	Rottweiler	M		17
12	Schnauzer	MN	6	16
CCLR				
1	Mixed	M	6	21
2	Pit-bull	F	5	16
3	Bull terrier	FS	5	29
4	Siberian H	M	4	32
5	Mixed	MN	7	25
6	Pitt-bull	M	5	14
7	Weimaraner	F	7	15
8	Labrador	M	5	23
9	Bull terrier	M	4	25
10	Mixed	F	5	20
11	Labrador	FS	5	16
12	Mixed	MN	6	18

*M* male, *MN* male, neutered, *F* female, *FS* female, spayed, *BCS* body condition score, *BSS* bioarth scale score

Inclusion criteria constituted the absence of any concurrent systemic or orthopedic disease, including a determination of hematologic, blood, and urine biochemical profiles, and the dog could not have received treatment of any kind since the previous month. A complete clinical evaluation (physical examination, including vital signs, neurologic, and orthopedic exams) assured that their specific joint OA was the only reason for the lameness.

- Group ED: To confirm or discard OA, standard radiographic views of both elbow joints were taken.
- Group CCLR: At physical examination, all dogs showed articular effusion of some degree and were positive for tibial compression and cranial drawer tests, which were done to assess the lack of stifle joint stability. Radiographs under tibial compression in recumbency confirmed the presence of unilateral CCLR.

Radiographs in all groups (included control group) were taken under sedation with dexmedetomidine IV 10 ± 20 μg/kg (Dexdomitor, Zoetis, Spain).

Additionally, the Bioarth score [13], a numeric rating scale based in radiological findings and joint functionality, was also reported Additional file 1.

### Pressure platform analysis

A pressure platform (Loran Engineering, Bologna, Italy) was placed, leveled, and aligned in the center of a 7 m runway. The device contained 2096 pressure sensors, consisting of 1cm^2^ distribution in an area of 48 × 48 cm. The range of pressure was 30-400 kPa with an acquisition frequency 100 Hz.

#### Posturographic exam

Dogs were placed in a square standing stance (with their limbs in a rectangular position and the head held directly in front), while the dog’s owner remained in front of the animal to attract the dog’s attention at a close distance. When the dogs seemed relaxed, data collection began and continued for 20 s at a sampling frequency of 100 Hz. In this way, statokinesiograms and stabilograms were obtained. Pressure distribution (%) between contralateral limbs, as in a static recording, was also obtained in this phase. Three valid trials were obtained from fore or hindlimbs depending on the study group and from each of the four limbs of the control dogs.

#### Dynamic Pedobarography

Dogs were leash guided by their owners when walking over the pressure platform. Walk velocity was measured through a motion sensor (Pasco, California, USA) positioned 1 m from the platform. Moreover, only those trials in which the animals walked in a narrow interval of velocity (1.2 ± 0.2 m/s) and acceleration (± 0.2 m/s2) were considered. Three valid trials for each dog were recorded at a sampling frequency of 100 Hz. A trial was considered valid when the studied limb fully supported over the pressure platform and when the dog walked next to the owner without pulling on the leash and without head turns. The pressure platform was interfaced with a dedicated computer using Biomech® (Loran Engineering, Bologna, Italy) software designed for the acquisition, storage, and graphic conversion of data. To avoid interference in measurements, this software allowed data to be discarded from those sensors that recorded different limbs within the same gait cycle of those studied.

Measured parameters with this technique were:

1. Paw area (cm^2^); The difference between lame and sound limbs was calculated using the following formula: % difference = 200 (A_CL_ - A_LL_)/(A_LL +_ A_CL_), where A_CL_ is the area of the sound limb in the study group or the limb with a higher value in the control group, and A_LL_ is the area of the lame limb in the study group or the limb with a lesser value in the control group.
2. Mean pressure (MP) (Kpa); The difference between lame and sound limbs were calculated in the same manner, that is, % difference = 200 (MP_CL_ - MP_LL_)/(MP_LL +_ MP_CL_).
3. Peak pressure (PP) (Kpa); similarly, the difference between the lame and sound limbs were calculated: % difference = 200 (PP_CL_ - PP_LL_)/(PP_LL +_ PP_CL_).

### Force platform analysis

A force platform (Pasco, California, USA) was placed adjacent to the pressure platform in such a way that recordings from animals were performed in the same session. DataStudio software (Pasco, California, USA) was used to obtain PVF (N) values from three valid trials. Mean values were normalized to body weight (%BW).

### Statistical analysis

For analyzing data, a linear mixed model was considered, being that the status “Study-Control” was a fixed effect and the dogs were random effects. The dogs were randomly selected from the population of sound and lame dogs, and the interest was to check the differences attributable to status. Normality was tested by the Shapiro-Wilk test and homoscedasticity by the Levene test. Significance level (alpha) has been established at 0.05, as usual. For statistical analysis, the R statistical environment version 3.4.0 was used (https://www.r-project.org/).

## Results

The mean (± SD) body weight of enrolled dogs was 38.3 ± 2.74 kg in the ED group, 36 ± 3.84 in the CCLR group, and 36.8 ± 3.44 kg in the control group. No statistical differences were found between groups (*P* ≥ 0.24). Mean age was 6 ± 2.23 in the control group, 5.6 ± 1.51 in the ED group, and 6 ± 1.87 in the CCLR group. No significant difference was found between groups (*P* ≥ 0.72).

In the following tables, the mean values ± SD, 95% confidence intervals, and *p*-values for the Shapiro-wilk (SWT) and levene (LT) tests of all obtained parameters are shown for ED (Table 2) and CCLR groups (Table 3), as well as reference values from Control group. In all cases, differences between LL and CL from ED and CCLR group were significant, and when compared with Control group, differences were also significant (*p* ≤ 0.02 and *p* ≤ 0.03, respectively). Differences between contralateral limbs in sound dogs were not significant in all cases (*p* ≥ 0.18). Data were all normal and homoscedastic.

**Table 2:** Posturographic,Dynamic Pedobarographic Parameters and PVF in ED Group, Expressed as Mean ± SD, and 95% Confidence Intervals, as well as *P*-values of t-test, Shapiro - wilk test and Levene test

			Difference	SWT	LT
Statokinesiogram (mm^2^)					
*Study*					
46.57 ± 22.47					
38.20, 54.95					
*Controls*					
2.29 ± 1.38			44.28 ± 3.76	0.21	0.98
1.66, 2.93			36.45,52.11		
Stabilogram (mm)					
*Study*					
X					
10.26 ± 4.14					
8.71, 11.82					
*Controls*					
3.14 ± 0.68			7.12 ± 0.72	0.16	0.98
2.75, 3.53			5.62,8.62		
*Study*					
Y					
1.70 ± 0.63					
1.46, 1.94					
*Controls*					
1.35 ± 0.5			0.35 ± 0.14	0.32	0.99
1.13, 1.57			0.05,0.65		
	LL	CL	% Difference		
Pressure distribution				*p* = 0.26	*p* = 0.96
*Study*	38.51 ± 3.70%	61.49 ± 3.70%	22.98 ± 7.40%		
	37.26, 39.76	59.77, 63.21	20.48, 25.49		
*Controls*	47.93 ± 1.16%	52.07 ± 1.16%	4.15 ± 2.33%		
	47.49, 48.36	50.30, 53.85	3.28, 5.02		
Paw Area (cm^2^)				*p* = 0.009	*p* = 0.56
*Study*	40.94 ± 3.70	51.97 ± 2.76	23.94 ± 9.97%		
	39.69, 42.19	50.81, 53.14	20.57, 27.31		
*Controls*	44.00 ± 2.82	47.20 ± 3.59	8.90 ± 6.64%		
	43.45, 46.35	47.04, 49.16	6.41, 11.38		
MP				*p* = 0.87	*p* = 0.72
*Study*	97.90 ± 12.99	149.69 ± 14.32	41.85 ± 18.94%		
	93.50, 102.29	143.88, 155.05	35.44, 48.26		
*Controls*	118.06 ± 9.19	130.15 ± 7.09	9.88 ± 8.70%		
	114.62, 121.49	127.51, 132.80	6.63, 13.12		
PP				*p* = 0.75	*p* = 0.25
*Study*	388.67 ± 33.41	461 ± 40.63	58.95 ± 40.78%		
	377.36, 399.97	448.21, 475.70	45.16, 72.75		
*Controls*	402.19 ± 42.03	435.51 ± 40.40	26.83 ± 30.43%		
	386.50, 417.88	420.42, 450.60	15.47, 38.20		
PVF				*P* = 0.05	*P* = 0.85
*Study*	62.29 ± 4.40%	74.33 ± 4.90%	12.04 ± 1.80%		
	59.95, 64.64	71.83, 76.83	(8.30, 15.77)		
*Controls*	68.85 ± 4.89%	69.56 ± 4.75%	0.71 ± 1.15) %		
	65.72, 71.98	66.39, 72.72	−3.02,1.61

**Table 3:** Posturographic and Dynamic Pedobarographic Parameters in CCLR Group, Expressed as Mean ± SD, and 95% Confidence Intervals

			Difference	SWT	LT
Statokinesiogram (mm^2^)					
*Study*					
11.91 ± 2.51					
10.95, 12.86					
*Controls*					
2.23 ± 0.71			9.67 ± 0.46	0.18	0.93
1.87, 2.60			(8.72,10.63)		
Stabilogram (mm)					
*Study*					
X					
7.60 ± 2.82					
6.54, 8.65					
*Controls*					
2.62 ± 0.60			4.97 ± 0.48	0.65	0.99
2.37, 2.87			3.96,5.98		
*Study*					
Y					
1.51 ± 0.56					
1.31, 1.72					
*Controls*					
1.00 ± 0.42			0.52 ± 0.12	0.61	0.97
0.82, 1.17			0.26,0.77		
	LL	CL	% Difference		
Pressure distribution				*p* = 0.85	*p* = 0.57
*Study*	21.21 ± 3.77%	78.79 ± 3.77%	57.59 ± 7.54%		
	(19.93, 22.48)	77.52, 80.07	55.04, 60.14		
*Controls*	47.77 ± 0.81%	52.23 ± 0.81%	4.45 ± 1.61%		
	47.77, 48.07	51.93, 52.53	3.85, 5.06		
Paw Area (cm^2^)				*p* = 0.93	*p* = 0.99
*Study*	27.50 ± 3.17	36.08 ± 3.46	27.10 ± 14.19%		
	26.43, 28.57	34.91, 37.25	22.30, 31.90		
*Controls*	31.63 ± 3.42	32.50 ± 2.43	2.98 ± 2.40%		
	30.36, 32.91	31.59, 33.41	1.65, 7.61		
MP				*p* = 0.16	*p* = 0.43
*Study*	137.84 ± 16.23	165.03 ± 13.51	18.19 ± 15.99%		
	132.35, 143.33	160.46, 169.60	12.78, 23.60		
*Controls*	155.40 ± 11.92	164.08 ± 14.48	5.30 ± 11.46%		
	150.95, 159.85	158.67, 169.48	1.02, 9.58		
PP				*p* = 0.59	*p* = 0.73
*Study*	367.00 ± 49.78	440.21 ± 42.12	48.06 ± 43.26%		
	350.16, 383.84	425.96, 454.46	33.42, 62.69		
*Controls*	401.13 ± 39.98	411.97 ± 55.35	7.50 ± 40.82%		
	386.20, 416.05	391.30, 432.63	7.74, 22.74		
PVF				*p* = 0.66	p = 0.93
*Study*	38.19 ± 4.52%	54.05 ± 5.34%	15.85 ± 1.89%		
	(35.64, 40.75)	(51.27, 56.83)	(11.93, 19.78)		
*Controls*	43.87 ± 3.62%	44.58 ± 3.88%	0.71 ± 0.53		
	(40.99, 46.75)	(41.71, 47.46)	(0,34, 1.77)		

**Fig. 1:**
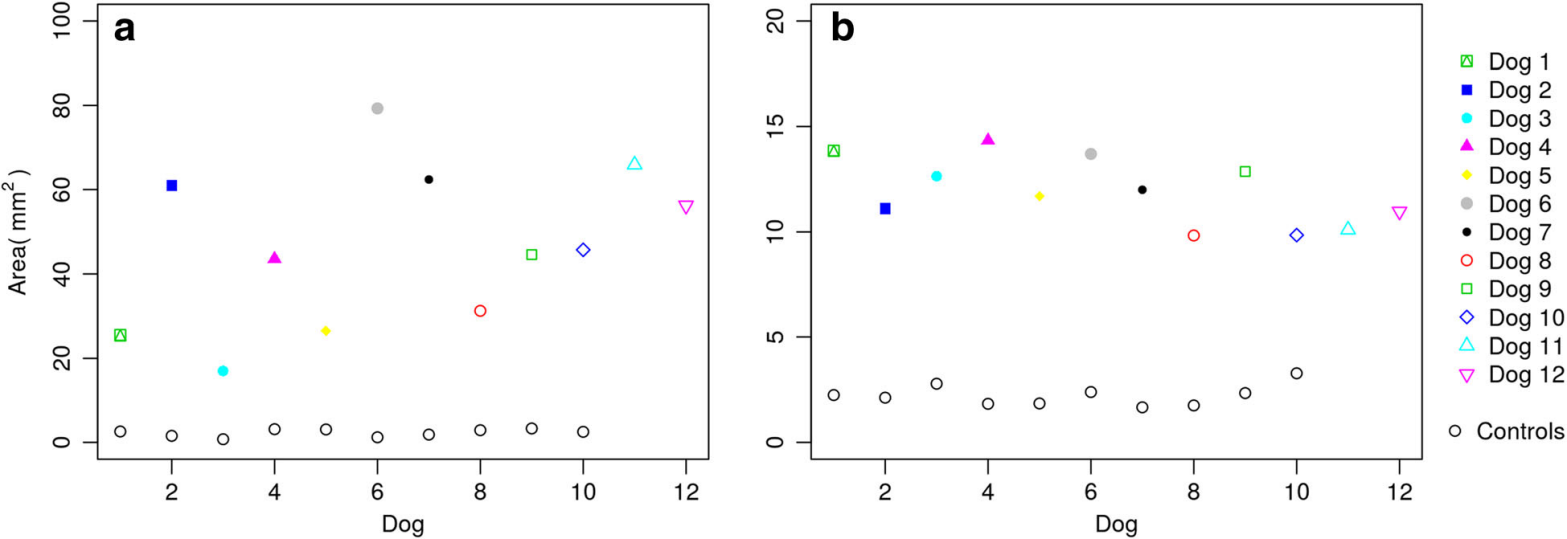
Individual Statokinesiogram Values Corresponding with ED (**a**) and CCLR (**b**) Dogs, Compared with Control Dogs. The ellipse area is always bigger in the study animals when compared with their respective controls

**Fig. 2:**
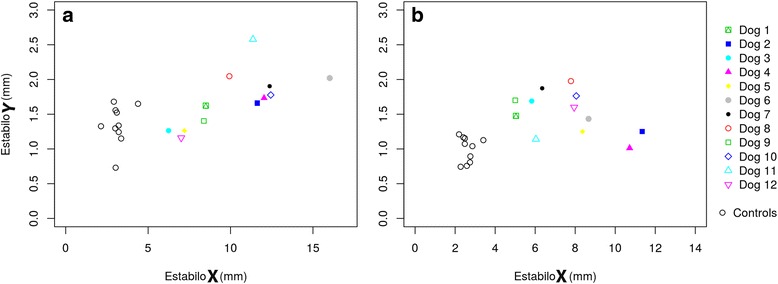
Individual Stabilogram Values Corresponding with ED (**a**) and CCLR (**b**) Dogs in both X-Y Axes, Compared with Control Dogs. All study dogs revealed a higher X displacement than the control dogs

**Fig. 3:**
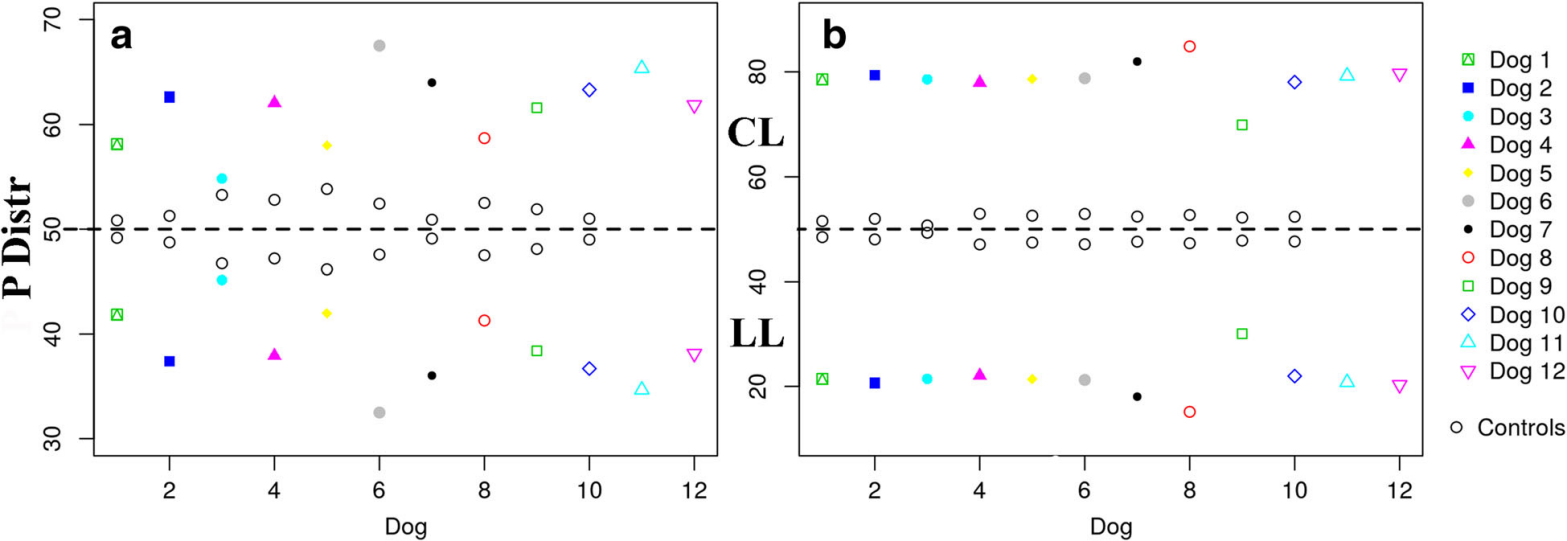
Pressure Distribution between Contralateral Limbs of All Dogs of ED (**a**) and CCLR (**b**) Dogs. LLs of each dog are under the dotted line, and CLs are above it. All dogs showed a higher asymmetry compared with the controls

**Fig. 4:**
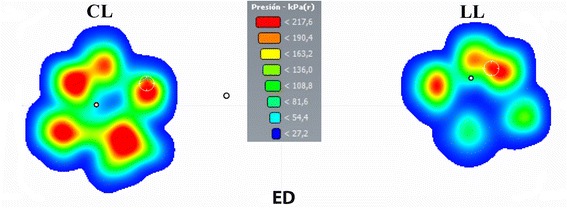
Pressure distribution color scale graphic of Sound (CL) and Lame (LL) Forepaws of Dog #2 from ED Group. In the sound limb, pressure distribution is symmetric, while, in the lame limb, highest pressure ranges (red, yellow colors) are only present craniomedially. Body COP (black circle) is displaced towards the sound limb

**Fig. 5:**
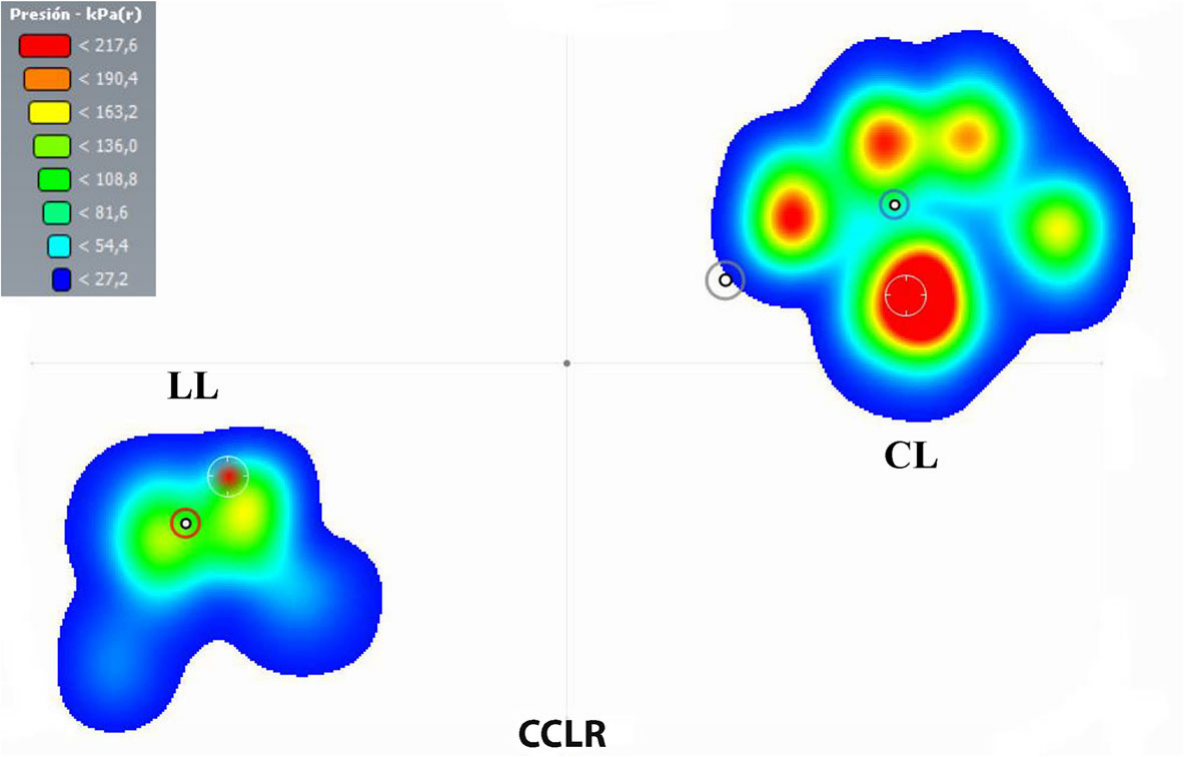
Pressure distribution color scale graphic of Sound (CL) and Lame (LL) Hindpaws of Dog #8 from CCLR Group. Antalgic posture can be seen with the lame limb caudally displaced. In the sound limb, highest pressure ranges (red, yellow colors) are found caudomedially and are almost inexistent in the lame limb. Body COP (black-grey circle) is displaced towards the sound limb

In the posturographic exam, data from statokinesiograms (Fig. 1a and b) and stabilograms (Fig. 2 a and b) showed significantly higher values in lame dogs of both study groups when compared with the control group, demonstrating a higher COP sway, or instability, in lame dogs. Pressure distribution values between LL and CL showed a clear asymmetry in both study groups, which is not the case of control dogs (Fig. 3 a and b). In addition to these results, the visualization of pressure ranges at standing revealed a medial migration of pressure in lame limbs in both the ED (Fig. 4) and CCLR (Fig. 5) groups.

**Fig. 6:**
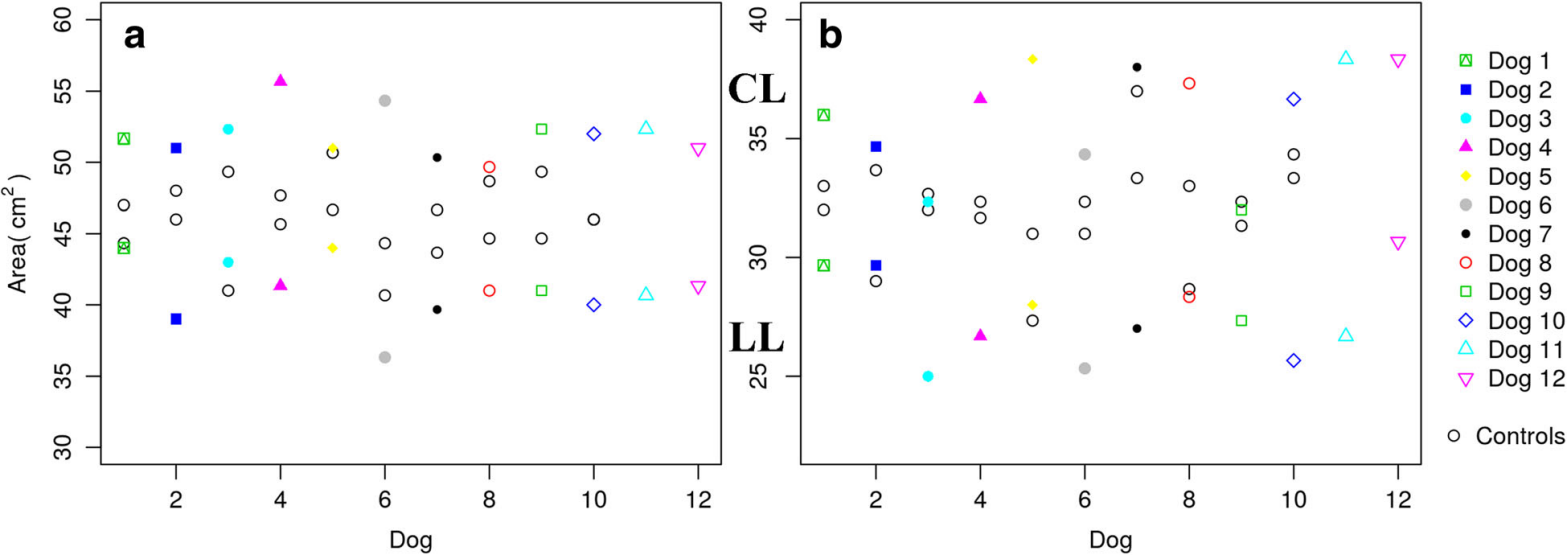
Paw area of LL and CL of All Dogs of ED (**a**) and CCLR (**b**) Dogs, Compared with Control Dogs. Differences between both contralateral limbs in lame dogs from both study groups are greater than those from control dogs

**Fig. 7:**
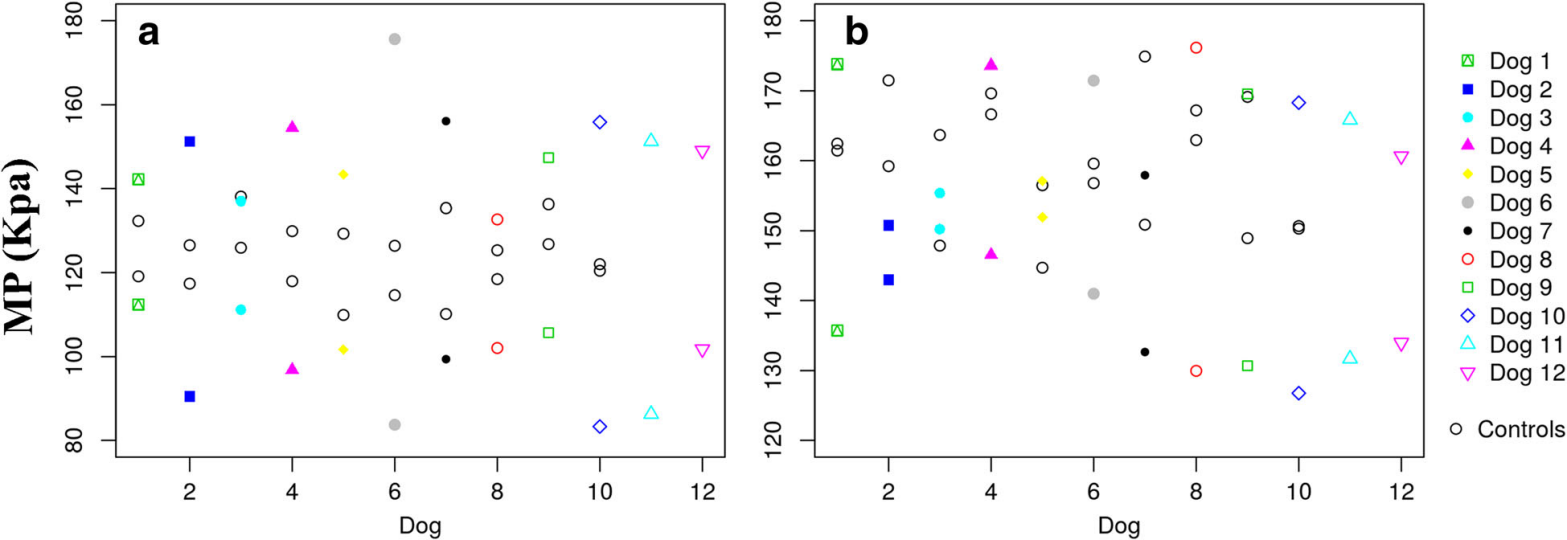
MP of LL and CL of All Dogs of ED (**a**) and CCLR (**b**) Dogs, Compared with Control Dogs. Differences between both contralateral limbs in lame dogs from both study groups are greater than those from control dogs

**Fig. 8:**
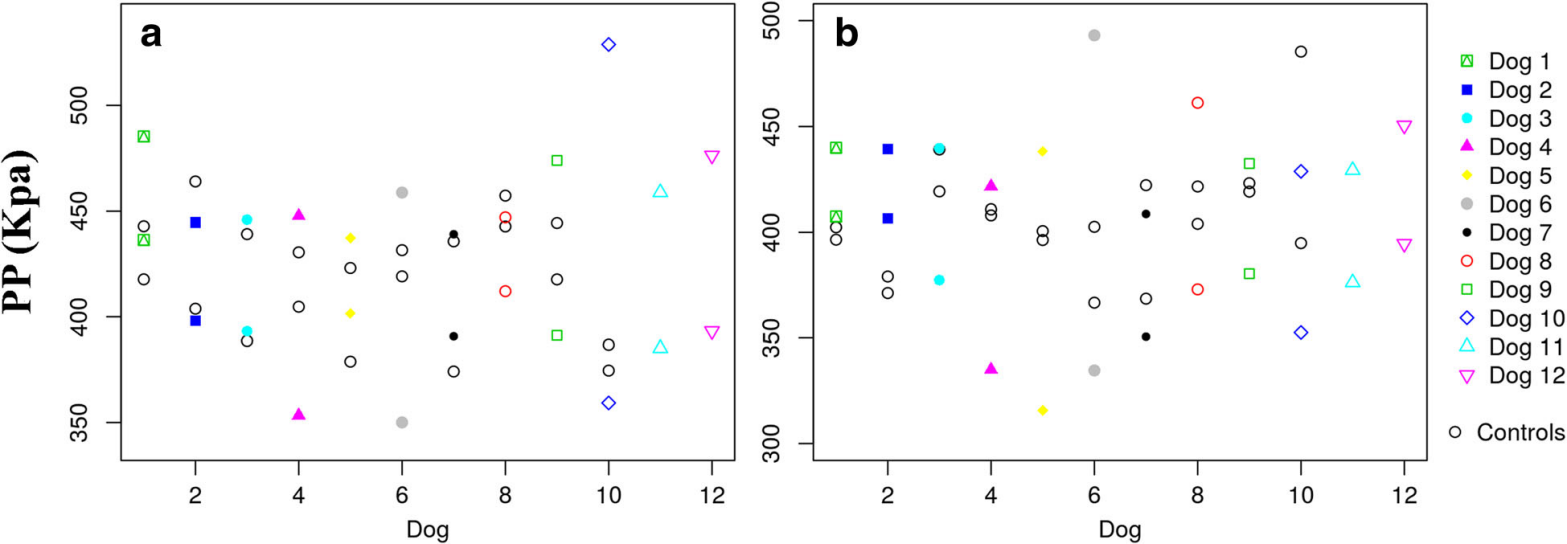
PP of LL and CL of All Dogs of ED (**a**) and CCLR (**b**) Dogs, Compared with Control Dogs. Differences between both contralateral limbs in lame dogs from both study groups are greater than those from control dogs

Regarding paw area, values were higher in sound limbs than lame limbs in both study groups, even compared with the control group (Fig. 6 a and b). In the same manner, MP and PP values were higher in sound limbs from the ED and CCLR groups, even when the control group is included in the comparison (Figs. 7 and 8, a and b). This fact is discussed below.

**Fig. 9:**
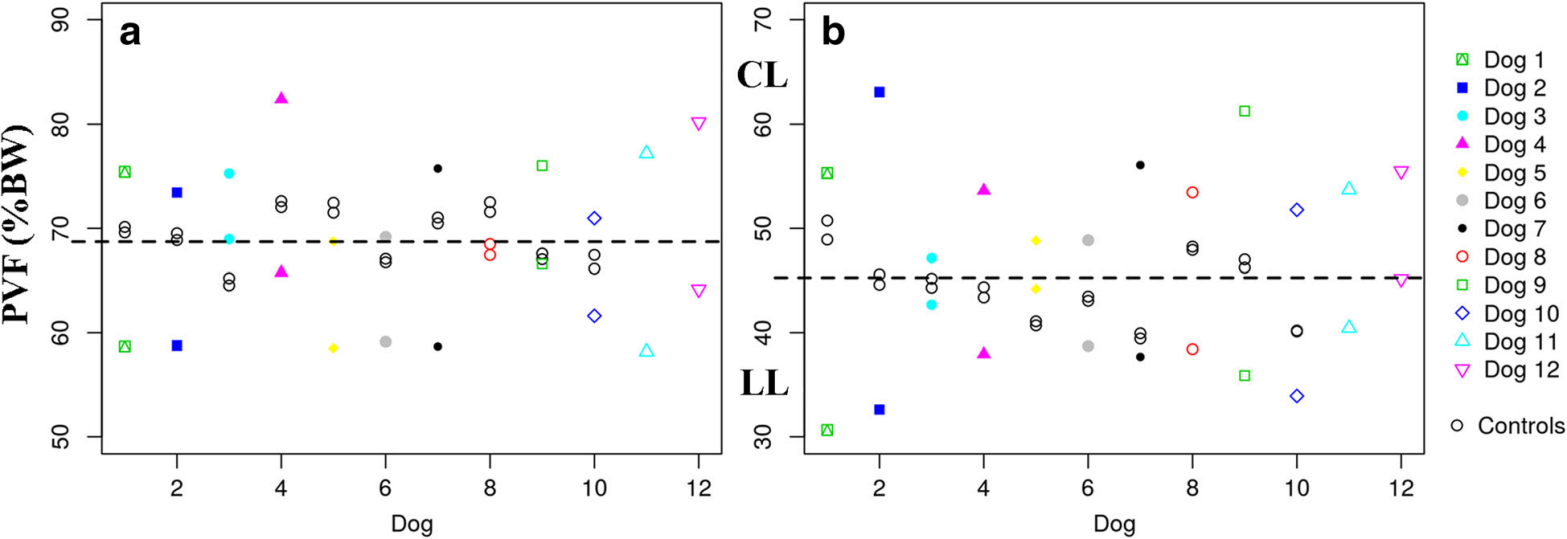
PVF of LL and CL of All Dogs of ED (**a**) and CCLR (**b**) Dogs, Compared with Control Dogs. Differences between both contralateral limbs in lame dogs from both study groups are greater than those from control dogs

**Fig. 10:**
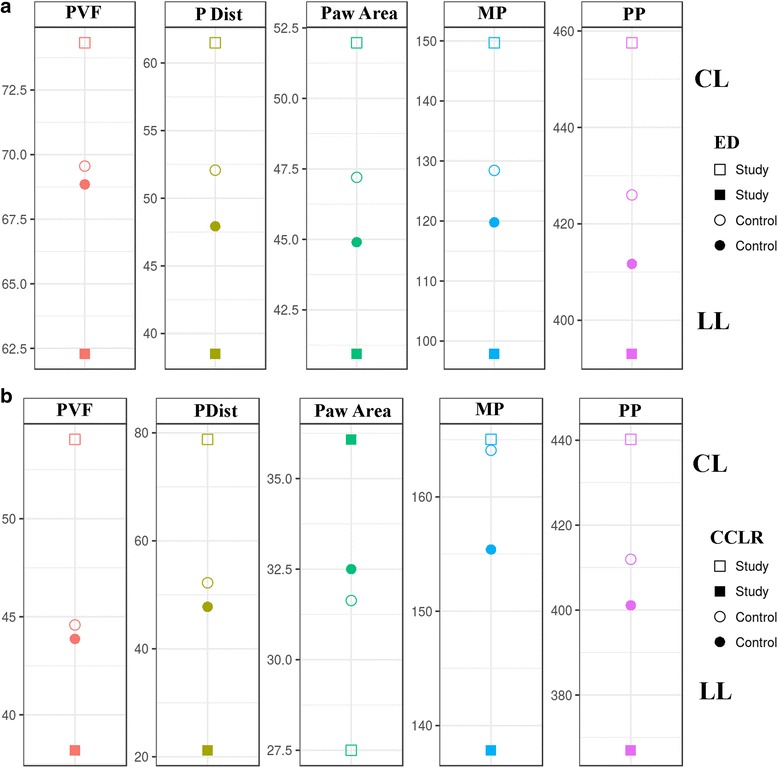
Comparison of Mean Values of all pedobarographic parameters of ED (**a**) and CCLR (**b**) Dogs with PVF. As can be seen, study groups (ED and CCLR) always show greater disparity (asymmetry) between contralateral limbs than the control group

PVF values showed a parallelism with those obtained with the pressure platform, with significant differences between LL and CL in the study groups and when compared with the control group (Figs. 9 and 10, a and b).

## Discussion

With the results presented, this study supports the hypothesis that significant differences in a set of postural and dynamic pedobarographic parameters between sound and OA dogs can be found. In the same manner, as in the last years, these differences have been detected and quantified with the gold standard test of limb function: the PVF [25]. Although some authors provide this parameter obtained with pressure platforms [27], recently published research reported some discordance with data obtained from force platforms [28]; these problems seem to be associated to calibration issues [29]. For that reasons, we preferred to obtain PVF with a force platform.

At the moment of redaction for the present study, only a report dealing with statokinesiography in dogs [19] has been found; on the other hand, pedobarographic studies are very scarce and limited to static recordings [20, 24, 30]. In contrast, multiple publications can be found regarding these techniques in human medicine, rehabilitation, and sport fields [31–33]. Based on our results, the use of pressure platform technology could, in the same manner, provide additional and complementary information and contribute to a more integrated study of lameness in dogs.

Concerning the posturographic exam, statokinesiograms and stabilograms showed significant differences between lame and sound groups, but when ED and CCLR groups are specifically compared, the mean statokinetic values from the ED group are much higher than the CCLR (46.57 vs 11.91 mm^2^), proving that, at least when these diseases are compared, imbalance is more accused in fore- than hindlimbs. This finding is aligned with some authors, which conclude that hindlimb lameness should be less noticeable as a lower proportion of body weight is supported by the hind limbs [34].

Compared with humans, this study with dogs reflects great differences in statokinesiograms, since the ellipse that contains COP migrations revealed a dominant laterolateral orientation as previously reported [19], whilst, in bipedal support of human species, the ellipse has its longer axe in the antero-posterior sense [35]. This fact could be explained because quadupedy in dogs implies in the stance a bigger distance between limbs in the craniocaudal axis than the laterolateral and, thus, a greater stability in the longitudinal sense. For that reason, the present study can also explain why in humans an ellipse area of statokinesiograms of about 100 mm^2^ in healthy subjects is considered normal [36] and, in contrast, this value is under 47 mm^2^ even in our lame dogs. This also explains why stabilograms from all dogs have lower values in Y-axis (craniocaudal) than X-axis values (laterolateral).

A symmetry index between contralateral limbs has been used in the last few years as a reliable parameter to assess lameness in both dogs and horses [2, 37, 38], where an index value of 0 represents a sound animal. Similarly, the pressure distribution difference between contralateral front and/or hindlimbs of 0% means a symmetric pressure distribution; therefore, the greater the difference, the greater the asymmetry. The differences in paw area between sound and lame limbs in both ED and CCLR study were significant, exhibiting how dog pads expand when submitted to pressure. However, in a previous study measuring paw area in sound dogs carrying dummies of various weights [2], no differences were found, which is in contrast with our results. Nevertheless, the carried weights in that study design may not have been enough to determine significant changes in this parameter. Furthermore, some authors have suggested that a dog’s pads spread differently as the response to pressure increases, indicating that the metacarpal pad is less responsive to pressure changes [20]. The present study only shows modifications in the global paw area in response to pressure; however, the study of different deformation (or even restitution) rates could potentially have clinical implications, which could be investigated in the future.

Greater disparity was found when MP differences between lame and sound limbs of the ED and CCLR groups were compared at a walk (41% vs 18%). The role of the head (and forelimb musculature) displacement as a counterweight when lame or sound horses and dogs move could explain this event [39]; this role can be breed-independent, according with previous studies [27]. PP differences between sound and lame groups were also significant; however, the high SD shown in the results prevent any conclusive interpretation. The cause of this fact should be elucidated in the future.

Although posturography and pedobarography have provided a set of useful parameters to detect both fore- and hidlimb lameness in dogs, this study has some limitations. First, middle to large dog breeds were used to clearly detect significant differences in some parameters, like MP, PP, or even paw area. Studies with smaller dogs would require higher resolution platforms. Second, the posturographic exam requires quiet, peaceful dogs, capable of maintaining the necessary posture during the recording time [19], which may be difficult to replicate. Finally, although compensatory pressure redistribution to the contralateral limb was detected and measured in both lame groups, the study did not include the homolateral limbs, which was previously reported in horses [39]; however, it would be interesting to include homolateral limbs in a further study.

## Conclusions

The set of data presented here suggest that posturographic and pedobarographic techniques may be promising tools to detect variations in COP sway characteristics, pressure distribution between contralateral limbs, paw area, MP, and PP of lame dogs with ED and CCLR.

## Additional file

Additional file 1: Bioarth scale for hip joints. (PDF 54 kb)

## Abbreviations

CL: is the area of the sound limb in the study groups or the limb with the higher value in the control group
COP: Center of pressure
LL: lame limb in the study groups or limb with lesser value in the control group
MP: Mean pressure
PP: Peak pressure
